# Big issues for small feet: developmental, biomechanical and clinical narratives on children’s footwear

**DOI:** 10.1186/s13047-018-0281-2

**Published:** 2018-07-06

**Authors:** Stewart C. Morrison, Carina Price, Juliet McClymont, Chris Nester

**Affiliations:** 1 School of Health Sciences, University of Brighton, 49 Darley Road, Eastbourne Campus, BN20 7UR UK; 2 Centre for Health Research, University of Salford, Salford, UK

**Keywords:** Children, Shoes, Foot development, Maturation, Footwear

## Abstract

The effects of footwear on the development of children’s feet has been debated for many years and recent work from the developmental and biomechanical literature has challenged long-held views about footwear and the impact on foot development. This narrative review draws upon existing studies from developmental, biomechanical and clinical literature to explore the effects of footwear on the development of the foot. The emerging findings from this support the need for progress in [children’s] footwear science and advance understanding of the interaction between the foot and shoe. Ensuring clear and credible messages inform practice requires a progressive evidence base but this remains big issue in children’s footwear research.

## Background

Children’s footwear is important for protecting feet [1] as children begin to advance their locomotor behaviours and explore and interact with their environment [2]. Development of the foot is ongoing throughout childhood [3] and this plasticity makes the size, shape and design of footwear important. These changes underpin the view that external factors, such as footwear choices, might influence the structural development and function of the foot, and impact on longer-term foot health [4–11]. However, evidence exploring this topic has been debated for decades [12–14] and still lacks scientific credibility [15]. Understanding the dimensions, design features and mechanical properties of children’s footwear are important but, in the absence of clear evidence, this remains a contentious topic. Existing views on children’s footwear have remained unchallenged for years and out-dated literature [9] continues to influence the contemporary clinical, commercial and industry narratives upon which footwear messages are based. There remains little understanding of the evolving role and meaning of footwear across childhood and adolescence [10] and this is key to repositioning the translation of age-appropriate, meaningful advice about footwear into clinical practice. There also exists the need to ensure that that footwear messages are credible and based on the best available evidence.

Dated messages about footwear and contemporary practices are intersecting and understanding the existing knowledge base is important. The following narrative review draws upon the developmental, biomechanical and clinical literature to synthesise existing knowledge about children’s footwear. Drawing these three narratives together allows us to identify areas where further work is indicated and form a clearer view of the priority messages for children and their parents/carers.

## Methods

A literature search was undertaken in October 2017 to retrieve articles related to the subject. PubMed, Google Scholar and Science Direct search engines were used, and pre-determined keyword searches included paediatric or children (‘s) or infant and footwear and shoes and foot; development; evolution; ethnicity; morphology; anatomy; biomechanics; down syndrome; cerebral palsy; disabilities; foot health; foot pain; function was undertaken. All articles were available in full-text and in English. Additional hand-searching of reference lists and conference proceedings was also undertaken to ensure that studies relevant to this work were identified and included. A narrative review of the studies was undertaken.

### Developmental perspective

Foot development is a complex interplay of intrinsic biological variables as well as extrinsic factors such as footwear and mechanical forces [3]. It is understood that the human foot did not evolve concurrent with footwear although archaeological evidence for footwear use dates to at least 30,000 years ago [16, 17] where some form of foot covering was needed for thermal insulation, as well as protection from injury [18]. The protective role of footwear for children is not debated but the impact on foot development remains uncertain [4, 19].

The effects of footwear on foot development have focussed primarily on the morphology of the medial longitudinal arch [4, 7, 8, 20, 21], although more recent studies are beginning to focus on the functional effects of footwear [4, 19, 22, 23]. Through measurement of static footprints, Echarri and Forriol [7] investigated the development of foot morphology in 1851 Congolese children aged 3–12 years and reported that footwear had little influence on morphological parameters of the feet. This contrasted with Rao and Joseph [8] who reported in their earlier study that footwear had a detrimental effect on the development of the medial longitudinal arch. This analysis of static footprints from 2300 children aged 4–13 years reported that flatfoot was more common in children who wore closed-toe footwear, and least common in the children who were unshod. Evidently, methodological limitations compromise the validity of these existing studies, but debate remains. Findings from a comparative analysis of 3-D foot morphology in 86 preschool and 419 primary school children from Australia compared against age, gender, height and BMI-matched German children [24] concluded that the German children had significantly longer and flatter feet compared to their Australian counterparts. A more recent study in habitually barefoot and habitually shod Kenyan children and adolescents [23] compared foot structure and function and reported that habitually barefoot children had greater foot [shortening] strength (*n* = 76) and barefoot children spent more hours per day engaged in physical activity (*n* = 62). The longer-term effects of habitual (barefoot running and) walking on biomechanical, health and motor performance outcomes remain to be determined [25]. Looking beyond morphology and anthropometric parameters is an essential shift in this area and recent work looking at the ontogenetic changes in foot strike patterns in early walking [26] highlighted the importance of understanding the structure-function relationships of the developing foot, as well as the impact of the foot-shoe interaction. Understanding how footwear and their materials (e.g. sole construction) respond to the demands of the growing foot [27, 28] and influence sensory stimuli and sensory and motor development [2, 29] are emerging topics which will help advance the debate in this area.

The breadth of studies looking at the impact of footwear on foot development are vast, but many are compromised by methodological limitations. It must be acknowledged that the sample sizes in some of the existing studies are considerable but the complexities of exploring developmental (e.g. cultural, ethnic, evolutionary) factors and their interactions are challenging, and this remains a considerable barrier to progress in this area.

### Biomechanical perspective

The influence of footwear on the biomechanical interaction between the foot and the environment is key to informing the therapeutic role of footwear in the management of foot problems in children. It also underpins the hypothesis that footwear might have a long-term effect on foot-function. It is clear from the developmental context that footwear choices in childhood are complex and whilst inappropriate choices have long been reported to impact on foot development and long-term foot health, there remains few studies which have explained such mechanisms [6, 8, 9]. Understanding the impact of footwear on foot development is also difficult due to the challenges in defining the foot as a functional biomechanical unit throughout childhood, as well as the challenges with conducting gait analysis in infants and children [30].

The biomechanical parameters most commonly reported in the literature are spatio-temporal and a recent meta-analysis [31] reviewed data from 11 publications spanning childhood (1.6–15 years of age). This analysis concluded that walking velocity, stride length, step length, stride time, base of support, double support and stance time all increased during shod walking, but cadence and single support time decreased. This supported earlier findings [32] where increases in gait velocity, step length and stride length were reported for a large cohort of school children (*N* = 656; aged 5–13 years) walking in trainers. Again, cadence was seen to reduce. Similarly, walking velocity in children walking barefoot in a laboratory environment was slower than when shod in a trainer (*N* = 20; 8–12 years; 1.33 m.s^− 1^ versus 1.41 m.s^− 1^) [33]. To walk at the same speed on a treadmill children demonstrated a longer stride length in a trainer compared to barefoot (1.03 m versus 1.00 m) [34]. These results do not appear to be consistent in earlier walkers where minimal differences in step length have been identified between shod and barefoot walking within the first 6 months of walking onset [35], however the footwear style utilised in this study was not described. This suggested that the mechanisms to cope with perturbations to standard gait occur later once gait is less variable and are more sensitive to factors such as footwear. All reported work has been undertaken in cultures where shod walking is commonplace and therefore the influence on habitually barefoot infants and children is unquantified.

Three-dimensional motion capture offers a gold-standard approach to gait analysis and could be key to helping us advance understanding of the influence of footwear on gait biomechanics in children. During shod walking (in trainers) a significant reduction in midfoot motion in all planes compared to barefoot waking has been reported [33], with considerable [72%] reduction in sagittal plane motion. Wegener et al. [31] hypothesised that this reduced the functional capability of the foot in terms of energy return. Alongside the reported kinematic changes, muscle activity is also thought to be influenced by footwear in children [36]. An increase in tibialis anterior activity in children wearing footwear, presumably to enable the floor clearance and/or because of the increased weight compared to the barefoot, has been reported. Footwear ultimately influencing the muscle activation of the lower limb is also inferred by increased ankle angle at initial contact in running shoes compared to minimalist shoes and barefoot conditions (*N* = 36; 6–9 years of age) [37]. Data from a small study (*N* = 14) looking at kinetics of gait reported that the age of a shoe could influence children’s running biomechanics, with older footwear resulting in higher loading rates (+ 23%, *p* = 0.016) [38]. Lower impact peaks are evident in barefoot running compared with shod however, minimalist footwear do not match this pattern and are not significantly different to cushioned footwear in children [37]. These results are also not mirrored if the children are forced to rear foot strike [39].

Existing advice about whether children are best suited to soft and flexible or hard and stiffer footwear has been debated for many years [9]. There is currently no evidence informing guidance on shoe stiffness with few studies exploring this. In one study, early walkers (*N* = 26; independently ambulating for 0–5 months) were found to have wider and shorter step length with reduced stance time when walking in soft, flexible footwear compared with stiffer shoe conditions [40]. However, there were no significant differences in the number of trips or falls between any of the shoe conditions in this study. In older children (*N* = 18, age = 8.2 ± 0.7 years) footwear designed to be lighter and more flexible had no significant effect on spatio-temporal parameters compared to the original design, although within this study changes in footwear stiffness were not objectively quantified and were assessed subjectively [41]. However, a significantly wider forefoot was recorded in the children when waking in the more flexible shoe. This research suggests that more flexible footwear inhibit the foot less and this could be considered important in infancy when the foot is developing in a non-systematic manner [42] and perhaps largely driven by external forces.

The existing literature suggests that footwear influences gait parameters but whether these effects are important in terms of function or longer-term foot health and development remains to be determined. Understanding the functional effects of footwear are important for bridging theory into practice but challenges with small sample size (s) are an existing limitation with the current literature.

### Clinical perspective

Footwear education and literacy is a core element of the clinical management of foot and lower limb complaints in children [11, 43] yet remains a challenge for many (clinicians and parents alike). Given the paucity of literature, footwear purchasing-habits and behaviours are poorly understood but remain important determinants of clinical intervention; the interaction between the foot, intervention (e.g. foot orthoses), and footwear choices are important for a positive outcome. Little is understood about the foot within the shoe [42, 44] and health-related trends (e.g. musculoskeletal symptoms in childhood obesity) expose the distance between existing research and current practice and highlight the need for greater understanding about the therapeutic potential of footwear choices.

The effects of footwear choices on functional outcomes and foot health remain poorly understood and there is a need to advance footwear research to better inform clinical practice [15]. Existing evidence highlights the importance of footwear for children with common foot pathologies such as calcaneal apophysitis [45], rheumatological conditions such as Juvenile Idiopathic Arthritis [46, 47], and genetic syndromes [48] but there are many gaps in existing evidence [49]. Down syndrome is a common intellectual disability and one of the few where research about footwear exists [48]. Musculoskeletal foot complaints in Down syndrome are common [50, 51] and footwear plays an important role in supporting the foot and helping children maintain mobility. The combination of peripheral joint hypermobility and low muscle tone results in a characteristic pes plano-valgus foot shape and makes difficulties with footwear fit very common [48]. Recent work in children and young people with down syndrome highlighted that poorly-fitting footwear (specifically footwear width) was associated with increased levels of foot-specific disability [48]. This is concerning given that foot specific disability has considerable impact on children’s function [52]. The therapeutic potential of footwear choices is important [48, 53–55] but many clinicians will understand the many struggles that children and their families face when looking to purchase footwear.

Understanding more about the social dimensions of footwear and what they mean to children is an important step forward that will help shape directions with footwear design, as well as ensuring that footwear advice in practice is meaningful. As a pre-requisite to effective conversations in practice about footwear choices for children, understanding what factors inform parent purchasing practices is important for clinicians to help understand how best to deliver age-appropriate footwear advice, and influence existing behaviours. Studies exploring attitudes towards children’s footwear are dated [12, 56] and theory in this area warrants review. The temporal landscape of footwear choices throughout childhood [57] is particularly important as children become more influential in their decision making, more responsive to fashion-trends and branding. Nevertheless, buying a child their first pair of shoe is a significant event for parents and families and this early decision making could help explain approaches to foot health behaviour throughout childhood. A greater awareness and consideration of the social and cultural aspects of footwear in our practice may be a simple strategy to improving the literacy in this area.

There is some indication in the existing literature that footwear is an important component of children’s foot health and a critical component of the clinical management of foot problems in children. To offer credible health messages about children’s footwear there must be a robust evidence-base to inform clinician’s advice and assist child and parent decisions about appropriate footwear choices. This remains an area where further research is needed and exposes how current practices are hampered by the lack of evidence.

## Conclusion

Footwear choices throughout childhood are important but the lack of empirical data about contemporary footwear science is a considerable issue. This review has exposed many weaknesses in our existing understanding of children’s footwear [practices and research] and supports the need to re-frame our thinking about footwear. It is time to move current debate away from the issues that have circulated for years and advance research exploring the developmental, biomechanical and clinical elements of children’s footwear. This will support advances with knowledge and underpin the integration of research data into clinical practice.
